# Violence and Suicidal/Nonsuicidal Self-Injury Among Adolescents Undergoing Residential Treatment: An Examination of the Predictive Validity of the SAVRY, START:AV, and VRS-YV

**DOI:** 10.1177/00938548231165531

**Published:** 2023-04-27

**Authors:** Andrew L. Gray, Jodi L. Viljoen

**Affiliations:** Université de Montréal; Simon Fraser University

**Keywords:** risk assessment, dynamic risk, adolescence, violence, mental illness, suicide, SAVRY

## Abstract

Using a retrospective study design, predictive validity of the Structured Assessment of Violence Risk in Youth, Short-Term Assessment of Risk and Treatability: Adolescent Version (START: AV), and the Violence Risk Scale–Youth Version (VRS-YV) was examined among 87 adolescents referred to a residential treatment program. With few exceptions, moderate to high accuracy was achieved for the three measures in predicting violence and suicidal/nonsuicidal self-injury occurring during the adolescents’ time in treatment. Accuracy of the measures peaked within 90 days for violence and gradually increased over the 180-day follow-up for suicidal/nonsuicidal self-injury. Dynamic factors were more predictive of repeated events involving violence relative to static/historical factors, whereas only factors from the START: AV were predictive of repeated events involving suicidal/nonsuicidal self-injury. These results emphasize the need for further examining the risk of adverse outcomes beyond violence among adolescents.

Adolescent risk assessment is an important and complex process, with mental health professionals having an ethical and professional duty to assess and manage risk. Despite substantial strides made in the development of adolescent risk assessment measures, much of what is known has been drawn from the psychiatric and psychological literatures with the primary focus being the assessment of risk for violence and general reoffending (Menon, 2013). Although adolescent violence remains an important area of consideration, assessing the risk of other adverse outcomes such as suicidal behavior and nonsuicidal self-injury (NSSI) is not only warranted by the empirical evidence but is also a requirement of mental health law (e.g., involuntary hospitalization) and many mental health agencies. Rates of suicidal behavior and NSSI are high among adolescents (Labelle et al., 2015), with suicide being the second most common cause of death worldwide among youth (Hawton et al., 2012).

Outcomes such as violence and self-injury are especially problematic among youth accessing residential treatment. For instance, among a large sample of children and adolescents undergoing residential (*n* = 9,942) and nonresidential (*n* = 525) treatment, those in residential care exhibited significantly higher pretreatment rates for behavioral problems (e.g., violence, aggression; 80.3% vs. 68.6%, respectively), self-injury (28.4% vs. 11.7%, respectively), and suicidality (i.e., ideation and attempts; 29.5% vs. 12.9%, respectively; Briggs et al., 2012). When compared to adults, children and adolescents treated within residential and in-patient settings have a much greater likelihood to harm themselves or others (Stewart & Hirdes, 2015), and those displaying increased levels of reactive aggression are at heightened risk to engage in suicidal behaviors (e.g., attempted or completed suicide; Hartley et al., 2018).

Such findings reinforce the need to identify adolescents at risk of adverse outcomes, particularly those within residential treatment settings; however, this area of research has remained largely unexamined. Although there is strong empirical support for assessing risk in adolescents using structured risk assessment measures (Viljoen, Gray, & Barone, 2016), many of the available measures meeting criteria for evidence-based tools are designed to assess risk of violence or reoffending (see A. L. Gray et al., 2019). As time and resources (e.g., specialized training) are required to administer a risk assessment measure, the question remains whether the information gathered (e.g., risk factor ratings) can help inform clinical decision-making regarding other adverse outcomes (e.g., NSSI). At present, there is a growing body of evidence suggesting substantial overlap among risk factors for various adverse outcomes (see Viljoen, Nicholls, et al., 2016, for a review). For instance, violence and suicide are known to coincide (e.g., murder-suicide; Douglas et al., 2013), and risk factors for violence such as impulsivity, childhood abuse/neglect, and previous self-harm have been found to be significantly predictive of subsequent self-injury/suicide attempts (e.g., Favril et al., 2020; K. R. Fox et al., 2015; McMahon et al., 2018). In predicting self-harm among adult forensic psychiatric samples, moderate to large effects have been observed across multiple research studies examining the accuracy of the Historical-Clinical-Risk Management-20 (HCR-20; Douglas et al., 2013), an adult risk assessment measure designed to assess risk of violence (Campbell & Beech, 2018; Daffern & Howells, 2007; Fagan et al., 2009; N. S. Gray et al., 2003; O’Shea et al., 2014).

This concept of overlapping risk factors across outcomes is best illustrated by the development of the Short-Term Assessment of Risk and Treatability (START; Nicholls et al., 2021; Webster et al., 2004), a structured professional judgment (SPJ) measure comprised of 20 dynamic factors that is designed to assess risk of multiple adverse outcomes (i.e., violence, nonviolent offenses, substance abuse, unauthorized absences, suicide attempt, NSSI, victimization, and health neglect). While meta-analytically examining the START, O’Shea and Dickens (2014) found that the strength and vulnerability scores were strong predictors of physical aggression but poorer predictors of self-harm. However, when examining the association between the SPJ-based risk estimates (hereafter referred to as risk judgments), they observed a larger effect size for self-harm and suggested that assessors may be taking into consideration only those “items most pertinent to the outcome in question” (O’Shea and Dickens, 2014, p. 998).

Existing evidence-based measures designed to assess risk of violence among male and female adolescents 12 to 18 years of age include the Structured Assessment of Violence Risk in Youth (SAVRY; Borum et al., 2006), the START: Adolescent Version (START: AV; Viljoen et al., 2014), and the Violence Risk Scale-Youth Version (VRS-YV; Wong et al., 2004-2011), with the three measures generally yielding moderate to large associations with violent reoffending (see Supplemental Table S1, available in the online version of this article). However, to our knowledge, no studies have examined the VRS-YV in predicting suicidal/nonsuicidal self-injury, and only a single unpublished study was identified for the SAVRY in predicting self-injury, resulting in a modest effect size (area under the curve [AUC] = .58). In contrast, four studies reported on the accuracy of the START: AV, an adolescent adaptation of the START, in predicting self-injury/NSSI. Results of these studies are mixed, with lower accuracy generally found for the vulnerabilities and strengths scores of the START: AV and slightly higher accuracy for the final risk judgments (see Supplemental Table S1, available in the online version of this article). Similar to the adult START, preliminary research with the START: AV has found differential associations between the individual items and the various risk judgments (Desmarais et al., 2012).

## The Present Study

With similar risk factors predicting violence and self-harm (e.g., impulsivity), it remains to be seen whether the SAVRY and VRS-YV, measures designed to assess violence risk, can predict outcomes beyond violence and general reoffending (e.g., NSSI) or whether the START: AV, a measure designed specifically to assess risk of multiple adverse outcomes (including violence, NSSI, and suicide attempt), will demonstrate greater predictive validity. To address this and contribute to the growing body of research on the generalizability of adolescent risk assessment, we examined the accuracy of the SAVRY, START: AV, and VRS-YV in predicting violence and suicidal/nonsuicidal self-injury (i.e., suicide attempt and NSSI) among a sample of adolescents admitted to a residential treatment facility using an archival, retrospective study design. It was hypothesized that the SAVRY, VRS-YV, and vulnerabilities/strengths scores of the START: AV would demonstrate moderate to high accuracy in predicting violence and low to moderate accuracy in predicting self-injurious behavior (i.e., suicide attempt, NSSI) occurring during the adolescents’ time in the residential treatment program. Regarding the risk judgments of the START: AV, we hypothesized they would be moderately to highly related to their respective domain.

A secondary theme of our study relates to the application of novel statistical approaches in further examining predictive validity. Although the AUC is the most common metric used to examine predictive accuracy in risk assessment research, much like logistic regression, reoffending status (i.e., whether a particular adolescent has engaged in violence) remains fixed over the entirety of the follow-up period (Heagerty et al., 2000). Variations in follow-up time at the individual level (i.e., censoring) are also ignored, with no information being provided as to the accuracy of a risk assessment measure over time. This has resulted in the development of time-dependant AUC analysis, which combines elements of survival analysis with AUC analysis. As applications of this method have begun to emerge (Glover et al., 2017; Viljoen et al., 2017), we sought to build upon the existing research by incorporating time-dependent AUC analysis to examine predictive validity at specific time-points and to determine the timeframe in which optimal predictive accuracy occurs or diminishes over time (i.e., the shelf life).

Despite the advantages of time-dependent AUC analysis and standard Cox regression, they remain limited in their application as they only account for time to an initial adverse outcome (e.g., first incident of violence at follow-up). As some adolescents may engage in or experience multiple adverse outcomes over the course of follow-up, there is a need for statistical approaches to account for these repeated occurrences. Although statistical approaches designed to examine repeated events exist (e.g., count data models such as Poisson or negative binomial regression) and have been applied to reoffending data (e.g., McLachlan et al., 2018; Walters, 2007), they do not account for time between events (Amorim & Cai, 2015). As a result, we examined repeated occurrences of adverse outcomes using recurrent event survival analysis (Kleinbaum & Klein, 2012).

## Method

Reporting of the methodology and results is in accordance with the Risk Assessment Guidelines for the Evaluation of Efficacy Statement (Singh et al., 2015). The statement consists of a 50-item checklist designed to increase consistency in reporting among risk assessment studies that examine predictive validity. Research approval for the current study was granted by the Office of Research Ethics of Simon Fraser University and the Applied Practice Research and Learning Branch of the Ministry of Children and Family Development of British Columbia, Canada.

### Participants

The current sample included 87 adolescents admitted to a residential treatment facility in Western Canada that provides specialized programs to adolescents with significant psychiatric and behavioral difficulties. All participants were referred to the facility through forensic psychiatric or community-based mental health services and received residential treatment in one of two programs. The behavioral program is an eight-bed residential treatment program designed for adolescents with mental health problems (typically consisting of conduct disorder with comorbid mental health disorders). This program assists caregivers and communities when substantial problems related to the provision of care exist and serves as the designated inpatient program for youth between the ages of 12 and 18 years who have been found unfit to stand trial (UST) or not criminally responsible on account of mental disorder (NCRMD). In contrast, the general program is a six-bed residential treatment program that incorporates assessment, intervention, and postdischarge services designed for youth experiencing internalized symptoms related to thought, mood, or anxiety disorders.

Approximately a third of the sample participated in the behavioral program (33.3%), with the remainder attending the general program (66.7%). Age at admission ranged from 12 to 18 years (*M* = 15.44, *SD* = 1.42), and the average length of stay within the program was 157 days (*SD* = 139.30, range = 73-846 days). Adolescents attending the two programs did not significantly differ with respect to age; however, those attending the behavioral program had significantly longer stays (*U* = 245.50, *p* < .001, *r_rb_* = .85) and were more likely to be male (χ^2^ [1] = 7.53, *p* = .006, *φ* = –.29). Most of the sample were born in Canada (90.8%; two cases were unknown), over half were male (55.2%), and the racial/ethnic composition was primarily European Canadian/White (55.2%) and Indigenous (24.1%). Status under the provincial Mental Health Act was available for 25 of the adolescents from the behavioral program and 47 from the general program, with a higher percentage of the behavioral program being admitted on an involuntary basis (80.0% vs. 6.4%, respectively).

### Measures and Adverse Outcomes

Interrater reliability could not be established for the following risk assessment measures due to a lack of resources, time constraints, and unavailability of trained research assistants at the time of data collection. In addition, site-specific factors such as restrictions on access to medical records, staffing changes, and the closing of the original treatment facility and subsequent relocation of the medical records further complicated the data-collection process. As such, we report on the interrater reliability reported in the peer-reviewed literature under each measure.

#### The Structured Assessment of Violence Risk in Youth (Borum et al., 2006)

The SAVRY is a 30-item SPJ guide designed to assess risk of future violence and assist in intervention planning/risk management in youth 12 to 18 years of age. It comprises 24 risk factors grouped into three risk domains (i.e., historical risk factors, social/contextual risk factors, and individual/clinical risk factors) and six protective factors representing a protective domain (i.e., protective factors). The SAVRY manual provides operational definitions and rating instructions, with risk factors being rated using a three-level coding structure (i.e., low, moderate, and high) and protective factors being rated dichotomously (i.e., present/absent). Item ratings on risk and protective factors are not summed for clinical purposes, rather evaluators use the SAVRY to make a Summary Risk Rating (SRR) of low, moderate, or high regarding an adolescent’s risk of violent reoffending. In addition to the SRRs, we calculated a risk total score and protective score by summing the 24 risk and six protective factors, respectively. A strong evidence base supports the interrater reliability and internal consistency of the SAVRY across research and applied settings, with the intraclass correlation coefficient (ICC) ranging from good to excellent for the SRR and risk total score of the SAVRY (see Borum et al., 2021; Koh et al., 2020).

#### The Short-Term Assessment of Risk and Treatability: Adolescent Version (Viljoen et al., 2014)

The START: AV is an SPJ guide designed to assess risk of various adverse outcomes (e.g., violence, victimization, NSSI) in youth 12 to 18 years of age. It comprises 24 items (with an optional case-specific item) falling into three clusters: individual adolescent, relationships and environment, and response to interventions. All items of the START: AV are rated as low, moderate, or high based on whether the youth has displayed minimal, some, or substantial strengths or vulnerabilities on a factor within the past 3 months, respectively. As such, a factor may simultaneously be considered both a strength and vulnerability (e.g., presence of prosocial and antisocial peers). Following the rating of the individual items, the presence of a prior history (i.e., any time prior to the past 3 months) or recent history (i.e., within the past 3 months) of an adverse outcome is determined, with a final risk estimate (hereafter referred to as risk judgment) of low, moderate, or high being made by the assessor for each of the nine adverse outcomes embedded within the START: AV. Consistent with prior research, the current study calculated total scores for the strengths and vulnerabilities subscales. Viljoen et al. (2012) found excellent interrater reliability for the strengths and vulnerabilities total scores (ICC [single raters] = .92 and .86, respectively), whereas ICC values ranged from good to excellent for the risk judgments (ICC [single raters] = .52 to .88).

#### The Violence Risk Scale–Youth Version (Wong et al., 2004-2011)

The VRS-YV is a 23-item clinician-rated risk assessment measure designed to systematically account for change in dynamic risk items and assess the extent to which an adolescent is at risk of committing a violent offense. Each of the items have been found to be empirically, conceptually, or theoretically related to violence in adolescents (Wong et al., 2004-2011). Items are rated on a four-point scale (0-3), with higher scores indicating increased risk levels. Dynamic items receiving a rating of 2 or 3 are considered criminogenic needs for which change is systematically rated using an adapted form of the stages of change. Stockdale et al. (2014) found the VRS-YV to have excellent interrater reliability (ICC = .87 for static, .89 for dynamic, and .90 for total score), whereas Koh et al. (2021) found good interrater reliability for the total score (ICC = .68). As the current study did not examine change in risk, only pretreatment (i.e., baseline) total, static, and dynamic scores were calculated, which is in keeping with prior validation studies examining the VRS-YV and related measures (e.g., Stockdale et al., 2014).

#### Adverse Outcomes and Time-at-Risk

Postbaseline adverse outcomes were coded based on available file information and only included incidents occurring during the adolescents’ time in program following the baseline assessment. As some adolescents, depending on privilege level, had access to the community (e.g., facilitated group outings, home visits) and grounds of the facility (e.g., attending school), incidents occurring both within and outside the facility were coded. Coding of the pre- and postbaseline adverse outcomes was based on definitions provided within the START: AV manual and consisted of a dichotomous (yes/no) variable, with a total number of incidents or count variable for postbaseline outcomes. Violence was defined as any attempted or actual physical harm committed against another (e.g., assault) and threats of death with or without a weapon in hand. Suicide attempt included any self-injurious behavior with expressed intent to die, regardless of the severity of the act (e.g., attempted suicide by hanging, severe slashing with expressed/confirmed suicidal intent). NSSI consisted of deliberate self-injurious behavior without the expressed intent to die (e.g., burning and/or slashing without evidence of suicidal intent). Time-at-risk was calculated for each of the adverse outcomes and represented the number of days between the end of the coding interval (i.e., baseline anchoring date) and either the date of the adverse outcome or date of follow-up/discharge (hereafter referred to as days-at-risk).

### Procedure

#### Inclusion Criteria

All clinical files closed between January 1, 2010, and June 20, 2014, were flagged for potential inclusion within the current study, with 167 independent files being identified. Two cases were unavailable for screening and removed from further analyses. To be included, the following criteria were required. First, to ensure that participants had an adequate follow-up period of at least 1 month, time from admission to discharge must have been ≥70 days, and they must have remained in the program ≥30 days following the intake assessment phase (using the date of the most recent report as an anchoring point). Second, to ensure sufficient information for scoring the risk assessment measures, a psychological assessment and family/psychosocial history report had to be available. Only reports completed through the residential program or forensic services just prior to or at the time of admission into the residential treatment program were considered as these reports were the most comprehensive and representative of the adolescents’ most recent functioning. For forensic referrals (i.e., adolescents deemed UST or NCRMD), a case management report and psychiatrist’s report to the provincial review board were required. Compared to the 78 adolescents not meeting inclusion criteria, there were no statistically significant differences observed between the 87 adolescents included within the study with respect to age (*U* = 3195.50, *r_rb_* = .06), gender (χ^2^ [1] = 0.03, *φ* = .01), race/ethnicity (χ^2^ [5] = 2.87, *ν* = .14; 11 cases were unknown), or program (χ^2^ [1] = 0.03, *φ* = –.01). Number of days in program was significantly shorter among the adolescents screened out (*M* = 76.14, *SD* = 89.24) relative to those who met inclusion criteria for the current study (*M* = 157.15, *SD* = 139.30, *U* = 1206.50, *p* < .001, *r_rb_* = .64).

#### Data Collection and Scoring of the Measures

Data collection and scoring of the measures was completed by a single rater (the lead author), a doctoral student in Clinical-Forensic Psychology at the time of data collection with clinical and research experience in scoring adult and adolescent risk assessment measures. To facilitate the reliable scoring of the SAVRY, START: AV, and VRS-YV, official training was received prior to data collection by one of the measure developers. Each of the trainings included the completion of a single or small number of practice cases compared against a gold standard scoring protocol. No structured risk assessment measure had been adopted into clinical use at the treatment facility during the period in which the files were closed or at the time of data collection. None of the files contained information on previously scored risk assessment measures.

Scoring of the study variables was based on a thorough review of psychological, psychiatric (including fitness/NCRMD assessment reports), medical, and social history and family assessment reports, in addition to discharge summaries contained within the closed health care files stored at the facility. Incident reports, progress notes, and any other pertinent documents available through the computerized information system were also reviewed. Other relevant documentation included completed psychological testing and questionnaires (e.g., cognitive testing, self-report measures), psychological/psychiatric reports prepared by forensic services, and other various documents available for adolescents with a history of involvement in the criminal justice system (e.g., nursing discharge summary from a forensic inpatient assessment unit). Although the date of the most recent assessment report served as the baseline anchoring date, when available, the date of the review board’s disposition hearing was used.

Due to the structural nature of the medical records and some portions of the files not being in chronological order, remaining blind to various adverse outcomes was not feasible when coding file information (e.g., recent police reports/charges were at times dispersed throughout earlier assessment reports). Although not regularly discussed in the literature, this is likely a common problem when coding medical records. To address this issue, special precautions were taken. Specifically, prior to initiating data collection, a file coding protocol canvassing the relevant domains (e.g., history of violence, substance abuse, mental health/cognitive state, leisure) was developed to enable recording of pertinent information required to score the risk assessment measures. Completion of the file coding protocol and coding of adverse outcomes occurred onsite, whereas the scoring of the risk assessment measures (i.e., item ratings and SRRs/risk judgments) occurred approximately 4 months later and was based on information contained within the file coding protocol. All measures were scored independent of the adverse outcomes previously coded from file, which were contained on a separate coding form, further reducing the risk of criterion contamination. The SRRs/risk judgments derived for the SAVRY and START: AV represent professional judgment ratings of the lead author and were made in accordance with instructions outlined within their respective manuals.

Scores on the SAVRY, START: AV, and VRS-YV were prorated when 10% or less of the items were omitted either due to a lack of information or lack of applicability, with no cases exceeding this threshold. Thirty-two percent of cases (*n* = 28) could not be rated on item D10 (insight into violence) on the VRS-YV due to having no known history of violence, whereas four cases could not be rated on item D14 (cognitive distortions) due to missing information (these latter cases were among the 28 cases that required the VRS-YV score to be prorated due to item D10). Although not a missing item per se, item 23 (medication adherence) on the START: AV was not applicable for 11.5% of cases (*n* = 10) due to the adolescents not being prescribed medication within the past 3 months. START: AV scores for these 10 cases were prorated to ensure congruence with scores derived for the remainder of the sample.

### Data Analysis

Descriptive statistics (i.e., means and standard deviations) and frequencies were computed for domain/total scores of the SAVRY, START: AV, and VRS-YV. As the data were not normally distributed, intercorrelations were calculated using Spearman’s rank-order correlation (*r_s_*). Between-group comparisons were conducted using Mann-Whitney *U-*tests and chi-square analyses with the nondirectional rank-biserial correlation (*r_rb_*; Kerby, 2014) and phi coefficient (*φ*) or Cramer’s V for variables with more than two categories, representing their respective effect sizes.

AUC of the receiver operating characteristic (ROC; Hanley & McNeil, 1982) was selected to examine predictive validity and was calculated using the R package “pROC” (Robin et al., 2011). AUC values can range from 0 to 1 (with .5 representing chance) and represent the probability that a randomly selected case will score higher on a risk assessment measure than a randomly selected control (Hanley & McNeil, 1982). According to Rice and Harris (2005), AUC values of .556, .639, and .714 are considered reflective of small, moderate, and large effect sizes, respectively. To determine the degree of association between the risk assessment measures and continuous outcome variables (i.e., total number of incidents and days-at-risk), Spearman’s rank-order correlation (*r_s_*) was calculated. Finally, to account for time to an adverse event, Cox proportional hazards survival analyses were conducted; however, we used the penalized version to reduce potential bias in the estimation of the hazard ratio (Heinze & Schemper, 2001) due to the smaller sample size and low base rates. Owing to the number of analyses, we provide a Bonferroni correction per statistical method (α = .05/19 = .003).

Time-dependent AUC (AUC_*t*_) analysis was conducted to examine predictive validity at specific time-points (e.g., 30, 90, and 180 days after baseline) and to determine the timeframe in which optimal predictive accuracy occurs or diminishes over time (i.e., the shelf-life; Heagerty et al., 2000). Combining elements of ROC and survival analysis, the time-dependent AUC represents the area under the time-dependent ROC curve and is defined as the probability that a risk score measured on a random case (e.g., an adolescent who has engaged in violence) exceeds that for a random control (e.g., an adolescent who has not engaged in violence) at time *t* (where *t* represents a fixed point in time—e.g., 60 days after baseline). Among the various forms of the AUC_*t*_ is the cumulative/dynamic time-dependent AUC (AUC_*t*_^*C/D*^) (Heagerty & Zheng, 2005). The AUC_*t*_^*C/D*^ is dynamic with respect to specificity such that if *T_i_* (i.e., the survival time for subject *i*) is greater than *t* for a case, then the case will serve as a control (i.e., if an identified adolescent who commits violence at follow-up had not been violent by time *t*, they would be counted as nonviolent at time *t*). However, once *t* ≥ *T_i_*, the individual is classified as a case (i.e., the adolescent is recognized as having committed violence). All initial events occurring throughout the time interval are included, with the base rate increasing over time as prior cases are retained in the calculation of sensitivity. As a nonparametric approach is recommended when calculating AUC_*t*_^*C/D*^ to ensure monotony of the ROC curve and to protect against dependence between the marker and censoring (e.g., if violence risk were associated with death [a censoring variable], this would indicate that there is dependence), the nearest neighbor estimate was applied (Akritas, 1994). Ninety-five percent confidence intervals for the AUC_*t*_^*C/D*^ values were calculated using the nonparametric percentile method with 2000 bootstrap replicates.

As some adolescents may experience repeated occurrences of an adverse outcome such as violence over the course of the follow-up period, predictive validity of the measures was further examined using recurrent event survival analysis. Recurrent event survival analysis uses the Cox proportional hazards model to assess the relationship between a predictor (or predictors) and the rate of occurrence of an event (i.e., an adverse outcome) while allowing for multiple events per subject. We applied robust variance estimation to account for dependency among multiple observations originating from a single subject (Castañeda & Gerritse, 2010; Kleinbaum & Klein, 2012). Recurrent event analyses were conducted using the R package “survival” (J. Fox & Weiberg, 2018) and are presented using the Prentice-Williams-Peterson Conditional Probability (PWP-CP) model (see Kleinbaum & Klein, 2012). Within the PWP-CP model, the presence of a prior event increases the likelihood of subsequent events occurring (i.e., outside of the first event, a subject is not assumed to be at risk of a subsequent event without having experienced a prior event). For example, an adolescent would not be considered at risk of engaging in a second act of violence during the follow-up period without them having previously engaged in an act of violence. As a result, recurrent events are stratified with the number of events per subject represented by a stratum variable.

## Results

### Sample Characteristics, Adverse Outcomes, and Intercorrelations

Fifty-four adolescents (62.1%) had some form of police contact documented in their file (e.g., being cautioned by police, arrested/detained, escorted to hospital), with 25 adolescents (28.7%) having at least one prior/current charge (*M* = 1.92 charges, *SD* = 5.63, range = 0-43). Reasons for police contact included aggression/violence without charges (*n* = 16), aggression/violence with charges (*n* = 23), nonviolent antisocial behavior without charges (*n* = 8), nonviolent antisocial behavior with charges (*n* = 4), and mental health crisis (*n* = 3). History of antisocial behavior was noted among 90.8% of the sample, with a significantly higher percentage observed among those admitted to the behavioral program relative to the general program (*n* = 29 [100.0%] vs. *n* = 50 [86.2%], respectively; 4.41, *p* = .036, *φ* = .23). Adolescents admitted to the behavioral program had a significantly higher likelihood of having been charged relative to those admitted to the general program (*n* = 23 [79.3%] and *n* = 2 [3.4%], respectively; χ^2^ [1] = 54.33, *p* < .001, *φ* = .79), whereas adolescents within the general program had significantly higher total scores on the SAVRY, START: AV, and VRS-YV at the *p* < .001 level. Despite only a quarter of the total sample (*n* = 22) being charged with a violent offense (e.g., assault, uttering threats), 85.1% (*n* = 74) were identified as having a prior history of violence, with 36.8% (*n* = 32) having a recent history of violence (see Table 1). Having a history of suicidal behavior and NSSI was also prevalent among the sample, with a larger proportion engaging in prior and recent NSSI (65.5% and 39.1%, respectively).

**Table 1:** Descriptive Statistics for the SAVRY, START: AV, VRS-YV, and Prebaseline and Postbaseline Adverse Outcomes (*N* = 87)

Measure and variable	*M*	*SD*	Range	*n*	%	Intercorrelations					
						2	3	4	5	6	7
SAVRY											
1. Protective	1.59	1.34	0-4			−.63	−.62	.69	−.38	−.59	−.58
2. Risk total score	25.17	9.01	7-41			−	.76	−.70	.79	.87	.90
START: AV											
3. Vulnerabilities	28.64	8.38	11-46				–	−.82	.48	.77	.76
4. Strengths	20.43	8.95	4-41					−	−.41	−.72	−.70
VRS-YV											
5. Static	5.47	2.95	0-12						-	.64	.74
6. Dynamic	29.29	13.35	4-57							-	.99
7. Total	34.77	15.42	4-66								-
Prebaseline adverse outcomes											
Prior history (prior to 3 months)											
Violence				74	85.1						
Suicide attempt				36	41.4						
NSSI				57	65.5						
Recent history (in past 3 months)											
Violence				32	36.8						
Suicide attempt				7	8.0						
NSSI				34	39.1						
Postbaseline adverse outcomes											
Violence				30	34.5						
Days-at-risk	58.03	61.75	1-409								
Number of incidents	1.05	2.54	0-19								
Suicidal/nonsuicidal self-injury				16	18.4						
Days-at-risk	97.11	136.76	2-818								
Number of incidents	0.30	0.73	0-4								

*Note*. All correlation coefficients significant at the *p* < .001 level. SAVRY = Structured Assessment of Violence Risk in Youth; START: AV = Short-Term Assessment of Risk and Treatability: Adolescent Version; VRS-YV = Violence Risk Scale–Youth Version; NSSI = nonsuicidal self-injury.

Postbaseline follow-up period for the current study ranged from 30 to 818 days (*M* = 111.69, *SD* = 139.62), with incidents occurring within the first 2 days of the follow-up period (see Table 1). Violence occurring postbaseline was recorded for just over a third of the sample (34.5%), whereas presence of suicide attempt and NSSI were relatively low (8.0% and 14.9%, respectively). As a result, we combined suicide attempts with NSSI to represent suicidal/nonsuicidal self-injury. This included any attempted suicide and/or any self-injurious behavior with or without the intent to die. Sixteen adolescents within the sample (18.4%) engaged in some form of suicidal/nonsuicidal self-injury over the follow-up period. Among the intercorrelations between the three measures, medium to large effect sizes (Cohen, 1992) were observed among the vulnerability factors of the START: AV and the risk domains/total scores of the SAVRY and VRS-YV (*r_s_* = .48 to .77, *p* < .001; see Table 1). A large and significant effect size was found between the strength/protective factors of the START: AV and SAVRY (*r_s_* = .69, *p* < .001), and medium to large inverse associations were observed between the risk domain/total scores of the three measures and the strength/protective factors (*r_s_* = −.38 to −.82, *p* < .001).

### Validity of Baseline SAVRY, START: AV, and VRS-YV in Predicting Violence and suicidal/nonsuicidal self-injury

AUC values for the SAVRY risk total score and SRRs fell within the moderate to large range as defined by Rice and Harris (2005) for both outcomes, whereas the protective factors were less associated with violence relative to suicidal/nonsuicidal self-injury (see Table 2). With the exception of the strengths subscale predicting violence and suicide risk judgment predicting suicidal/nonsuicidal self-injury, AUC values observed for the START: AV exceeded the threshold for a large effect (AUC ≥ .71; Rice & Harris, 2005). With respect to the VRS-YV, the dynamic and total scores were significantly predictive, with large effect sizes being observed for violence and moderate effect sizes for suicidal/nonsuicidal self-injury. The static score, however, was not strongly associated with either outcome. Relative to the AUC analyses, similar trends were observed among the results of the penalized Cox regression analyses for violence but were less consistent for suicidal/nonsuicidal self-injury.^1^

**Table 2:** Predictive Validity Analyses for Violence and Suicidal/Nonsuicidal Self-Injury (*N* = 87)

Measure and variable	AUC	95% CI_AUC_	*r_s_* ^Time^	*r_s_* ^Incident^	Penalized Cox Proportional Hazards Model				
					*B*	*SE*	*χ* ^2^	HR	95% CI_HR_
SAVRY									
Violence									
Protective^a^	.60	[.48, .73]	−.01	−.20	−.17	.15	1.52	0.84	[0.63, 1.11]
Risk total score	.65*	[.53, .77]	−.06	.28**	.04	.02	4.17*	1.04	[1.00, 1.09]
SRR (violence)	.77***	[.68, .87]	−.16	.49***	1.03	.28	16.51***	2.81	[1.67, 5.11]
Suicidal/nonsuicidal self-injury									
Protective^a^	.76***	[.64, .88]	−.09	−.35***	−.64	.27	7.44**	0.53	[0.29, 0.85]
Risk total score	.65*	[.51, .79]	.24*	.19	.03	.03	1.13	1.03	[0.98, 1.09]
SRR (violent)	.74***	[.62, .86]	.26*	.34**	.82	.41	5.04*	2.28	[1.10, 5.53]
START: AV									
Violence									
Vulnerabilities	.71***	[.59, .83]	−.11	.39***	.07	.02	8.19**	1.07	[1.02, 1.12]
Strengths^a^	.70***	[.59, .81]	.10	−.35***	−.06	.02	7.44**	0.94	[0.90, 0.98]
Risk judgment (violence)	.79***	[.69, .88]	−.15	.53***	.93	.25	17.30***	2.53	[1.61, 4.27]
Suicidal/nonsuicidal self-injury									
Vulnerabilities	.73***	[.63, .84]	.26*	.31**	.06	.03	3.08	1.06	[0.99, 1.13]
Strengths^a^	.73***	[.62, .84]	−.17	−.32**	−.06	.03	4.10*	0.94	[0.88, 1.00]
Risk judgment (suicide)	.75***	[.62, .88]	−.08	.41***	1.19	.34	11.94***	3.30	[1.70, 6.43]
Risk judgment (NSSI)	.64*	[.51, .78]	.02	.23*	.45	.31	2.43	1.58	[0.89, 3.03]
VRS-YV									
Violence									
Static	.58	[.45, .70]	.01	.15	.05	.06	0.70	1.05	[0.93, 1.19]
Dynamic	.73***	[.62, .84]	−.10	.44***	.04	.01	9.36**	1.04	[1.02, 1.07]
Total	.71***	[.60, .83]	−.08	.41***	.03	.01	7.79**	1.03	[1.01, 1.06]
Suicidal/nonsuicidal self-injury									
Static	.51	[.34, .68]	.23*	−.01	−.06	.09	0.54	0.94	[0.79, 1.11]
Dynamic	.67*	[.53, .80]	.37***	.21	.01	.02	0.61	1.02	[0.98, 1.06]
Total	.65*	[.51, .79]	.36***	.19	.01	.02	0.28	1.01	[0.98, 1.04]

*Note.* AUC = area under the curve; CI_AUC_ = confidence interval of AUC; *B* = regression coefficient; *SE* = standard error of *B*; HR = hazard ratio; CI_HR_ = confidence interval of the HR; SAVRY = Structured Assessment of Violence Risk in Youth; SRR = Summary Risk Rating; START: AV = Short-Term Assessment of Risk and Treatability: Adolescent Version; NSSI = nonsuicidal self-injury; VRS-YV = Violence Risk Scale–Youth Version.

a For ease of interpretation, scores on the protective/strengths domains were reversed for the AUC analysis such that higher scores represent a deficit in protective factors.

* *p* < .05. **p < .01. ***p < .001 (two-tailed test). Bonferroni correction: *p* = .003.

Although the SAVRY, START: AV, and VRS-YV demonstrated statistically significant associations with the number of incidents of violence, the three measures tended to be unrelated to days-at-risk for violence. In contrast, significant associations with days-at-risk were observed for suicidal/nonsuicidal self-injury; however, these tended to be in the opposite direction of what would be expected (e.g., greater days-at-risk for adolescents rated as higher risk). Moreover, while the SAVRY and START: AV demonstrated significant associations, the VRS-YV was unrelated to the number of incidents of suicidal/nonsuicidal self-injury during the follow-up period.

#### Time-Dependent AUC Analysis

Despite some fluctuations in AUC values at the outset of the follow-up period, which was truncated at 180 days as only 15 adolescents remained under observation beyond this point, there was a gradual increase in the AUC_*t*_^*C/D*^ values for the three measures in predicting violence (see Supplemental Figure S1, available in the online version of this article). This appeared relatively consistent for the initial 100 days, with some of the scores declining sharply in their predictive accuracy shortly thereafter. This trend was reflected in the AUC_*t*_^*C/D*^ values for the measures at *t* = 30, 90, and 180 days (see Table 3), with the largest values being produced at *t* = 90 days. Regarding suicidal/nonsuicidal self-injury (see Table 3, Supplemental Figure S2, available in the online version of this article), the VRS-YV displayed poor predictive accuracy at the outset of the follow-up period. Nevertheless, there was a gradual increase in AUC_*t*_^*C/D*^ values for the VRS-YV beyond the initial 50 days, with the static, dynamic, and total scores achieving similar results, after which the static score gradually declined to some extent. A gradual increase was also evident for the SAVRY risk total score and START: AV in predicting suicidal/nonsuicidal self-injury, with the AUC_*t*_^^C/D^^ values ranging from .65 to .77 at *t* = 180 days. In contrast, the protective factors domain and SRR of the SAVRY displayed relatively consistent predictive accuracy over the follow-up period.

**Table 3:** Cumulative/Dynamic Time-Dependent AUC Values for Violence and Suicidal/Nonsuicidal Self-Injury (*N* = 87)

Measure	Violence (AUC_*t*_^*C/D*^)			Suicidal/nonsuicidal self-injury (AUC_*t*_^*C/D*^)		
	*t* = 30 days	*t* = 90 days	*t* = 180 days	*t* = 30 days	*t* = 90 days	*t* = 180 days
SAVRY						
Protective^a^	.54 [.41, .67]	.63 [.48, .76]	.61 [.49, .75]	**.71 [.54, .86]**	**.71 [.60, .86]**	**.71 [.61, .87]**
Risk total score	.63 [.47, .75]	**.69 [.54, .79]**	.24 [.16, .76]	.49 [.31, .66]	.61 [.47, .73]	**.65 [.51, .79]**
SRR (violence)	**.74 [.62, .84]**	**.78 [.66, .88]**	.75 [.14, .88]	**.72 [.52, .87]**	**.69 [.53, .82]**	**.73 [.57, .86]**
START: AV						
Vulnerabilities	.60 [.49, .74]	**.71 [.60, .83]**	.56 [.40, .81]	.58 [.43, .70]	**.69 [.56, .78]**	**.74 [.61, .83]**
Strengths^a^	**.62 [.51, .74]**	**.69 [.57, .81]**	.35 [.19, .78]	.63 [.49, .76]	.63 [.49, .75]	**.68 [.55, .82]**
Risk judgment (violence)	**.71 [.60, .82]**	**.81 [.70, .89]**	.**80 [.70, .89]**			
Risk judgment (suicide)				**.72 [.51, .89]**	.67 [.48, .86]	**.77 [.57, .93]**
Risk judgment (NSSI)				.57 [.36, .75]	.61 [.44, .76]	**.68 [.52, .83]**
VRS-YV						
Static	.59 [.44, .70]	.63 [.48, .74]	.26 [.11, .71]	.41 [.18, .61]	.48 [.30, .66]	.44 [.24, .68]
Dynamic	**.63 [.51, .76]**	**.74 [.62, .84]**	.57 [.43, .82]	.51 [.32, .65]	.65 [.48, .75]	**.70 [.55, .81]**
Total	.63 [.50, .74]	**.73 [.58, .83]**	.47 [.26, .81]	.48 [.30, .62]	.63 [.47, .73]	**.67 [.53, .81]**

*Note.* The corresponding 95% confidence intervals for the AUC_*t*_^^C/D^^ values are provided in square brackets and were calculated using the nonparametric percentile method with 2000 bootstrap replicates. Bolded values are significant at the *p* < .05 level. AUC = area under the curve; AUC_*t*_^^C/D^^ = cumulative/dynamic time-dependent AUC; SAVRY = Structured Assessment of Violence Risk in Youth; SRR = Summary Risk Rating; START: AV = Short-Term Assessment of Risk and Treatability: Adolescent Version; NSSI = nonsuicidal self-injury; VRS-YV = Violence Risk Scale–Youth Version.

a For ease of interpretation, scores on the protective/strengths domains were reversed for the time-dependent AUC analysis such that higher scores represent a deficit in protective factors/strengths.

#### Recurrent Event Survival Analysis

Among the 30 adolescents who engaged in violence during the follow-up period, a total of 91 incidents were recorded. For suicidal/nonsuicidal self-injury, 26 incidents were recorded among 16 adolescents. As time-dependent AUC and standard survival analysis do not account for recurrent events, we next examined whether the SAVRY, START: AV, and VRS-YV could predict repeated events of violence and, separately, suicidal/nonsuicidal self-injury while controlling for the order of events and days-at-risk between events using recurrent event survival analysis (Table 4). Except for the static domain of the VRS-YV, results of the PWP-CP model revealed that greater levels of assessed risk were associated with an increased likelihood of recurrent episodes of violence. Although the strengths domain of the START: AV was a statistically significant predictor of recurrent episodes of violence, the protective factors score on the SAVRY was unrelated. Regarding suicidal/nonsuicidal self-injury, only the START: AV was predictive of recurrent episodes.^2^

**Table 4:** Conditional Recurrent Event Survival Analysis for Violence and Suicidal/Nonsuicidal Self-Injury (*N* = 87)

Measure and variable	PWP-CP model					
	*B*	*SE_R_*	Wald	*p* Value	HR	95% CI_HR_
SAVRY						
Violence						
Protective	−.07	.08	0.65	.420	0.93	[0.79, 1.10]
Risk total score	.03	.01	5.65	.017	1.03	[1.01, 1.05]
SRR (violence)	.88	.22	15.58	< .001	2.41	[1.56, 3.73]
Suicidal/nonsuicidal self-injury						
Protective	−.36	.39	0.86	.354	0.70	[0.32, 1.50]
Risk total score	.01	.02	0.34	.560	1.01	[0.97, 1.06]
SRR (violence)	.19	.34	0.33	.566	1.21	[0.63, 2.33]
START: AV						
Violence						
Vulnerabilities	.05	.02	8.92	.003	1.05	[1.02, 1.09]
Strengths	–.04	.01	9.70	.002	0.96	[0.93, 0.98]
Risk judgment (violence)	.93	.22	18.60	< .001	2.53	[1.66, 3.87]
Suicidal/nonsuicidal self-injury						
Vulnerabilities	.07	.02	8.11	.004	1.07	[1.02, 1.12]
Strengths	−.07	.02	8.31	.004	0.93	[0.89, 0.98]
Risk judgment (suicide)	1.32	.23	33.81	< .001	3.73	[2.39, 5.81]
Risk judgment (NSSI)	.69	.25	7.46	.006	2.00	[1.22, 3.29]
VRS-YV						
Violence						
Static	.04	.04	0.89	.345	1.04	[0.96, 1.12]
Dynamic	.05	.01	23.25	< .001	1.05	[1.03, 1.07]
Total	.04	.01	20.34	< .001	1.04	[1.02, 1.06]
Suicidal/nonsuicidal self-injury						
Static	−.10	.08	1.38	.240	0.91	[0.77, 1.07]
Dynamic	.01	.02	0.26	.610	1.01	[0.98, 1.04]
Total	.00	.01	0.04	.841	1.00	[0.97, 1.03]

*Note.* PWP-CP = Prentice-Williams-Peterson Conditional Probability Model; *B* = regression coefficient; *SE_R_* = robust standard error of *B*; HR = hazard ratio; CI_HR_ = confidence interval of the HR; SAVRY = Structured Assessment of Violence Risk in Youth; SRR = Summary Risk Rating; START: AV = Short-Term Assessment of Risk and Treatability: Adolescent Version; NSSI = nonsuicidal self-injury; VRS-YV = Violence Risk Scale–Youth Version.

Bonferroni correction: *p* = .003.

## Discussion

### Prevalence of Violence and Suicidal/nonsuicidal self-injury

We sought to contribute to the growing body of empirical research on assessing risk in adolescents by examining the predictive validity of the SAVRY, START: AV, and VRS-YV among a sample of adolescents with significant psychiatric and behavioral difficulties. Unlike past research which has primarily focused on predicting violence and general antisocial behavior among justice-involved adolescents, our study focused on the prediction of violence and suicidal/nonsuicidal self-injury which are common among adolescents undergoing residential treatment (Briggs et al., 2012). Within the current sample, a relatively large number of the adolescents engaged in suicidal behavior and NSSI before entering the program (44.8% and 66.7%, respectively). Having a history of violence was also high among the sample (85.1% for prior history) despite less than a quarter having been charged with a violent offense. This latter finding emphasizes the importance of not solely relying on criminal history (e.g., charges) to determine whether there is a history of violence, particularly among adolescents. The degree of discrepancy between the rate of violent charges relative to the rate of violent behaviors within the current sample may be the result of attempts by families and caregivers to manage their children’s violent behavior within the home and/or with the aid of community agencies (e.g., group homes, mental health agencies), while also diverting them away from the criminal justice system through the use of other corrective means (e.g., suspensions, involuntary psychiatric admissions). Moreover, for a small number of the participants, their violent behavior may have occurred before the age in which they could be criminally charged.

Moderate prevalence rates were evident for postbaseline violence (e.g., 34.5%), whereas postbaseline rates for suicidal/nonsuicidal self-injury tended to be modest (e.g., 8.0% for suicide attempt, 14.9% for NSSI). Nevertheless, similar posttreatment rates have been found among youth in residential care (i.e., 35.2% for behavior problems, 19.7% for self-injury, and 14.5% for suicidality; Briggs et al., 2012), whereas among justice-involved adolescents, prevalence rates were lower relative to the current sample (i.e., 1.6% for suicide attempt and 11.4% for NSSI; Viljoen et al., 2012). In contrast, De Beuf et al. (2023) found much higher rates of self-injury and violence (41.5% and 73.6%, respectively) and lower rate of suicidal behavior (3.8%) among a Dutch sample comprised of 106 adolescents in medium- and high-security treatment units. Although such variation in prevalence rates across studies may be reflective of differences in sample characteristics (e.g., major mental illness), setting (e.g., residential treatment setting vs. community supervision), and study design (e.g., retrospective vs. prospective, self-report vs. staff recorded), they nevertheless highlight the need for professionals to assess and manage risk not only for violence and antisocial behavior but also for a broader range of adverse outcomes, particularly those more common among adolescents (e.g., NSSI). This ensures a more comprehensive assessment of risk which, in turn, can aid in appropriate resource allocation and matching with risk management/reduction efforts (A. L. Gray et al., 2019).

### Predictive Validity of the SAVRY, START: AV, and VRS-YV

With respect to predictive validity, our results lend preliminary support to their utility in predicting violence and suicidal/nonsuicidal self-injury among adolescents undergoing residential treatment. Although SAVRY and VRS-YV exhibited significant predictive accuracy for violence across all analyses, there was less consistency in their ability to predict suicidal/nonsuicidal self-injury, particularly repeated incidents. Although SAVRY and VRS-YV appear to be operating as intended (i.e., assessing risk of violence), this raises the possibility that there may be less overlap between risk factors for certain adverse outcomes (e.g., NSSI and those for violence) as contained within the two measures. The general lack of association with recurrent suicidal/nonsuicidal self-injury and the SAVRY and VRS-YV challenge, to some degree, the empirical findings with the HCR-20 (e.g., O’Shea et al., 2014) and may be reflective of differences in item content (e.g., greater focus of the HCR-20 on symptoms of major mental illness) and populations for which they were designed (adult vs. adolescent), or that the overlap in risk factors for violence and self-harm may be more prominent in adults as opposed to adolescents.

Concerning the START: AV, our results revealed it to be the most robust and consistent predictor of violence and suicidal/nonsuicidal self-injury, with vulnerabilities and strengths remaining predictive irrespective of statistical analysis. Although this latter finding runs contrary to research conducted using the adult START (e.g., O’Shea & Dickens, 2014), this may reflect differences in item content, with adjustments to item anchors to increase relevance with outcome and developmental considerations being taken into account during the development of the START: AV. Our results for the START: AV are promising, and clinical adoption of the measure may assist mental health professionals in carrying out their ethical and professional duty to assess and manage risk of violence and self-harm. Arguably, the START: AV has the potential to inform comprehensive care while increasing efficiency and reducing resources required to clinically assess multiple adverse outcomes through the use of a single risk assessment measure. The START: AV may also serve to complement existing measures such as the SAVRY and VRS-YV in light of its focus on short-term risk for multiple adverse outcomes. As it is common practice to administer both the HCR-20 and START in adult forensic settings (O’Shea et al., 2014), the START: AV could be administered in tandem with a measure of longer-term risk that incorporates static/historical risk factors. Moreover, the robust findings related to the strengths/protective factors of the START: AV and SAVRY contribute to the growing body of research on the importance of assessing protective factors among adolescents (e.g., Dickens & O’Shea, 2018).

Unlike previous research (e.g., Viljoen et al., 2017) which has found an inverse association between risk scores and days-at-risk (e.g., higher-risk youth reoffend at a faster rate), days-at-risk were found to be either unrelated (for violence) or demonstrated an association in the opposite direction of what was expected (for suicidal/nonsuicidal self-injury). This counterintuitive finding may be reflective of a greater degree of supervision or restriction being placed on high-risk/high-need adolescents when entering the program, which may have inhibited their ability to engage in adverse outcomes such as suicide attempt and NSSI. This in turn may also explain the inverse association between days-at-risk and strengths on the START: AV and, to a lesser extent, the protective domain of the SAVRY, as those exhibiting a greater number of strengths may have been monitored less.

Contrary to previous research findings that predictive validity remains stable over time (Glover et al., 2017; Viljoen et al., 2017), analyses conducted using time-dependent AUCs revealed that, with few exceptions, optimal predictive accuracy for SAVRY, START: AV, and VRS-YV in predicting violence generally peaked within the initial 3 months, with some scores exhibiting sharp declines in accuracy thereafter. Although this aligns with the rating timeframe specified when scoring the START: AV, this finding was unexpected regarding the SAVRY as there is neither an explicit timeframe provided when rendering a final judgment of risk, nor was its predictive validity found to diminish over a 2-year follow-up period (Viljoen et al., 2017). One sample-specific factor that may have impacted our results is that higher-risk adolescents remained in treatment longer than their lower-risk counterparts, which may have resulted in marker-dependent censoring (i.e., loss of follow-up for some adolescents due to being discharged on account of their low-risk status). Although marker-dependent censoring was accounted for when calculating the time-dependent AUC values, this may still have impacted our results due to the relatively small sample size (Kamarudin et al., 2017). Another possible explanation may relate to the treatment context as adolescents within the current sample may have become more stabilized with the passage of time, thus diminishing the clinical relevance of their assessments over the follow-up period; however, this runs contrary to the findings regarding suicidal/nonsuicidal self-injury. As such, further research examining the timeframe for optimal predictive accuracy under various conditions is required before any firm conclusions are drawn.

### Strengths and Limitations

There are several methodological strengths associated with the current study. While studies examining the validity of the SAVRY, START: AV, and VRS-YV have used criminal justice samples and outcomes primarily related to violence and antisocial behavior, the current study also examined suicidal/nonsuicidal self-injury among a sample of adolescents undergoing residential treatment for significant psychiatric and behavioral difficulties. In considering the sample and setting in which the current study was conducted, further insight is provided into the “behavior” of these measures beyond standard criminal justice settings and, to some extent, their generalizability to adolescents with complex mental health needs.

Furthermore, this study aimed to use novel data analytic approaches by employing statistical techniques not commonly found within the forensic psychological/psychiatric literature (e.g., cumulative/dynamic time-dependent AUC and recurrent event survival analyses). Despite the repetitive nature of adverse outcomes such as violence, very few studies have examined the rate of reoffending, and among those that have, the statistical approaches selected have often ignored survival time (e.g., negative binomial regression). To our knowledge, this study represents the first application of recurrent event survival analysis to the prediction of adverse outcomes such as violence. The application of recurrent event survival analysis has important implications to the field as it is more statistically powerful, better accounts for the realities of outcome studies (i.e., repeated occurrences), and represents a step forward in the evolution of risk assessment research.

Although promising, consideration of study limitations is warranted when interpreting these results. One such limitation is the lack of interrater reliability analysis for SAVRY, START: AV, and VRS-YV. Due to the nature of the file coding procedures and circumstances surrounding data collection, establishing interrater reliability with an independent rater was not feasible. However, official training provided by one of the developers was secured for each measure before commencing data collection. Prior research examining the interrater reliability of these risk assessment measures have generally found ICC values ranging from good to excellent among trained raters (e.g., Viljoen et al., 2012), and great care was taken in ensuring that the scoring of the risk assessment measures was in keeping with the scoring procedures as outlined within their respective manuals.

Although identified and acknowledged at the outset of data collection, the inability to remain blind to outcome when coding file information is a considerable limitation of the current study and may have inadvertently introduced bias into the results. Issues related to criterion contamination are not uncommon among archival/retrospective risk assessment studies (e.g., Edens et al., 2001), and various steps were taken to reduce the risk of contamination (e.g., use of file coding protocols). The use of separate file coding protocols to code outcome and risk-relevant information, combined with a 4-month delay, served to offset the likelihood of inadvertently linking coded outcomes with a given case when scoring the risk assessment measures. Nevertheless, application of interrater reliability analysis, use of multiple independent raters to separately score the risk assessment measures and code outcome, and/or having the files prepared in advance of data collection to ensure blindness to outcomes may have assisted in further reducing this risk.

Another limitation includes sample size as this may have resulted in power limitations for various analyses (e.g., AUC analysis). Efforts were made, however, to mitigate this using nonparametric analyses and other novel statistical approaches intended to reduce the impact of small sample sizes and low base rates (e.g., penalized Cox regression; Gibbon & Chakraborti, 2011; Kleinbaum & Klein, 2012). With the application of recurrent event survival analysis, more information was used, and greater statistical power was provided, thus yielding a more robust analysis of the predictive validity of the SAVRY, START: AV, and VRS-YV. Although smaller samples resembling the one used within our study are not uncommon within the adolescent risk assessment literature (e.g., McLachlan et al., 2018; Viljoen et al., 2012), this precluded us from conducting subsample analyses based on gender and race/ethnicity as samples below 50 can detrimentally impact the accuracy of the AUC estimates (Hanczar et al., 2010). Examining the effects of gender and race/ethnicity on predictive validity represents an important area in need of further research (Muir et al., 2020).

Other important limitations include combining adolescents from the two programs into a single sample for analyses and the retrospective nature of the study. Despite being housed within a single residential treatment facility with centralized services, meaningful differences remain between the two programs and the adolescents attending them as observed within the current study. Although combining adolescents from the two programs into a single sample impacted the results to some degree as evidenced by the AUC analysis for the general program, the variation in results does not appear to have been detrimental to the findings originating from the overall sample given its composition (i.e., all adolescents had significant psychiatric and behavioral difficulties, with 90.8% having a history of antisocial behavior and 62.1% having some form of police contact documented on file). Regarding the retrospective nature of the study and our subsequent reliance on archival data, coding of the risk assessment measures and outcomes was based on information recorded by staff at the facility. Reliance on archival data may be susceptible to the recording practices unique to the setting (Nicholls et al., 1999). As coding of the outcome data did not incorporate all potential methods of data collection (e.g., adolescent self-report), relying heavily on information recorded by staff, it is likely that the true prevalence rates were underestimated (Douglas & Ogloff, 2003). That said, the use of real-world clinical data has important implications in research and should be taken into consideration when balancing the methodological strengths and limitations of the current study.

### Summary and Future Directions

In summary, the current study provides a more comprehensive picture of the predictive validity of the SAVRY, START: AV, and VRS-YV. Although our results lend preliminary support to the generalizability of these measures extending beyond criminal justice samples to adolescents with complex mental health needs, future research would benefit from the use of a prospective study design conducted across various settings (e.g., general psychiatric, forensic psychiatric, and correctional) with interrater reliability analysis, multiple sources for outcome coding (i.e., adolescent/caregiver self-report, official records, and hospital charts), and an increased follow-up period to examine both short- and long-term validity. This would serve to increase the generalizability of the findings while also broadening the research base on adolescent risk assessment, further aligning research with clinical practice.

## Supplemental Material

sj-docx-1-cjb-10.1177_00938548231165531 – Supplemental material for Violence and Suicidal/Nonsuicidal Self-Injury Among Adolescents Undergoing Residential Treatment: An Examination of the Predictive Validity of the SAVRY, START:AV, and VRS-YV Click here for additional data file. Supplemental material, sj-docx-1-cjb-10.1177_00938548231165531 for Violence and Suicidal/Nonsuicidal Self-Injury Among Adolescents Undergoing Residential Treatment: An Examination of the Predictive Validity of the SAVRY, START:AV, and VRS-YV by Andrew L. Gray and Jodi L. Viljoen in Criminal Justice and Behavior

## Notes

1. AUC analysis is provided in the Supplemental Table S2 (available in the online version of this article) for adolescents within the general program (*n* = 58). Decreases in predictive accuracy were evident when examining violence, with some scales showing a marked decrease (i.e., risk total score of the SAVRY, vulnerabilities score of the START: AV). SRR of the SAVRY and strengths and violence risk judgment of the START: AV remained significant predictors, whereas the dynamic and total scores of the VRS-YV yielded nonsignificant AUC values falling just below the threshold for a moderate effect. With the exception of the static score of the VRS-YV, all measures demonstrated an increase in predictive accuracy for suicidal/nonsuicidal self-injury. Caution is warranted, however, in interpreting these results due to the small sample size.

2. As the number of recurrent events may cause the estimates to become unstable within the PWP-CP model, the data were truncated to three recurrent events (Amorim & Cai, 2015); however, the results did not differ substantially from those reported in Table 4. Results are available upon request.
