# Supplementary material for: Violence and Suicidal/Nonsuicidal Self-Injury Among Adolescents Undergoing Residential Treatment: An Examination of the Predictive Validity of the SAVRY, START:AV, and VRS-YV
Source: Crim Justice Behav. 2023 Apr 27;50(7):931–52. doi: 10.1177/00938548231165531 (PMC10265301; doi:10.1177/00938548231165531)
Supplement: sj-docx-1-cjb-10.1177_00938548231165531 – Supplemental material for Violence and Suicidal/Nonsuicidal Self-Injury Among Adolescents Undergoing Residential Treatment: An Examination of the Predictive Validity of the SAVRY, START:AV, and VRS-YV [file sj-docx-1-cjb-10.1177_00938548231165531.docx]

**Supplemental Materials**

**Table S1**

*Outcome Studies Examining Predictive Validity of the SAVRY, START:AV, and VRS-YV for Violence and Self-Injurious Behavior*

| Instrument and study | Domain | Outcome | *n* | Statistic | Results |
| --- | --- | --- | --- | --- | --- |
| SAVRY |  |  |  |  |  |
| Guy (2008) | Protective^c^ | Physical aggression^d^ | 615 (*k* = 7) | AUC*w* [95% CI] | .73 [.62, .85] |
|  | Risk total score | Physical aggression^d^ | 720 (*k* = 8) |  | .75 [.67, .82] |
|  | SRR | Physical aggression^d^ | 493 (*k* = 5) |  | .79 [.69, .89] |
| Koh et al. (2020) | Risk total score | Violent reoffending | 66 – 712 (*k* = 26) | AUC | .54 – .84 |
|  | SRR | Violent reoffending | 66 – 712 (*k* = 26) |  | .56 – .86 |
| Olver et al. (2009) | Risk total score | Violent reoffending | 1,032 (*k* = 9) | *r_w_* [95% CI] | .30 [.24, .36] |
| Viljoen et al. (2015) | Risk total score | Self-injury | 89 | AUC (SE) | .58 (.08) |
| START:AV |  |  |  |  |  |
| De Beuf et al. (2021) | Vulnerabilities | Physical aggression | 106 | AUC [95% CI] | .61 [.48, .73] |
|  |  | Self-injury | 106 |  | .48 [.37, .60] |
|  | Strengths^c^ | Physical aggression | 106 |  | .63 [.50, .76] |
|  |  | Self-injury | 106 |  | .44 [.33, .55] |
|  | Risk judgement | Physical aggression | 106 |  | .71 [.60, .82] |
|  |  | Self-injury | 106 |  | .68 [.57, .59] |
| Sher et al. (2017) | Vulnerabilities | Physical aggression | 90 | AUC [95% CI] | .70 [.59, .81] |
|  |  | Self-harm | 90 | *r* | .13 |
|  | Strengths^c^ | Self-harm | 90 | AUC [95% CI] | .63 [.52, .75] |
|  |  |  | 90 | *r* | -.08 |
| Viljoen et al. (2012)^a^ | Vulnerabilities | Violent reoffending | 73 | AUC (SE) | .70 (.07) |
|  |  | NSSI | 61 |  | .70 (.08) |
|  | Strengths^c^ | Violent reoffending | 73 |  | .73 (.08) |
|  |  | NSSI | 61 |  | .46 (.12) |
|  | Risk judgement | Violent reoffending | 73 |  | .65 (.07) |
|  |  | NSSI | 61 |  | .67 (.11) |
| Viljoen et al. (2015) | Vulnerabilities | Violent reoffending | 89 | AUC (SE) | .67 (.07) |
|  |  | Self-injury | 89 |  | .67 (.07) |
|  | Strengths^c^ | Violent reoffending | 89 |  | .70 |
|  | Risk judgement | Violent reoffending | 89 |  | .72 (.07) |
|  |  | Self-injury | 89 |  | .71 (.09) |
| VRS-YV |  |  |  |  |  |
| Koh et al. (2022)^b^ | Total (pre) | Violent reoffending | 233 | AUC | .63 |
| Lovatt et al. (2022)^b^ | Static | Violent reoffending | 256 | AUC [95% CI] | .60 [.53, .67] |
|  | Dynamic (pre) | Violent reoffending | 256 |  | .68 [.62, .75] |
|  | Total (pre) | Violent reoffending | 256 |  | .67 [.60, .73] |
| Stockdale et al. (2014) | Static | Violent reoffending | 145 | AUC [95% CI] | .77 [.69, .84] |
|  | Dynamic (pre) | Violent reoffending | 145 |  | .75 [.67, .83] |
|  | Total (pre) | Violent reoffending | 145 |  | .77 [.70, .85] |

*Note.* SAVRY = Structured Assessment of Violence Risk in Youth; VRS-YV = Violence Risk Scale-Youth Version; START:AV = Short-Term Assessment of Risk and Treatability: Adolescent Version; NSSI = Non-suicidal self-injury; AUC*w* = weighted AUC; AUC = area under the curve; CI = confidence interval; *r_w_* = mean weighted correlation; *r* = correlation coefficient; SE = standard error.

^a^ Sample sizes estimated using percentages provided in the text.

^b^ AUC values reported for Koh et al. (2020b) and Lovatt et al. (2022) are for 3-year and 5-year follow-up periods, respectively.

^c^ Scores on the Protective/Strengths domains were reversed in original publications such that higher scores represent a deficit in protective factors.

^d^ Physical aggression includes sexual violence.

**Table S2**

*Predictive Validity Analyses for Violence and Suicidal/Non-Suicidal Self-Injury for Adolescents Within the General Program (N = 58)*

| Measure and variable | AUC | 95% CI_AUC_ |
| --- | --- | --- |
| SAVRY |  |  |
| Violence |  |  |
| Protective^a^ | .51 | [.31, .72] |
| Risk total score | .56 | [.37, .74] |
| SRR (violence) | .73** | [.58, .89] |
| Suicidal/non-suicidal self-injury |  |  |
| Protective^a^ | .83** | [.61, 1.00] |
| Risk total score | .67 | [.44, .90] |
| SRR (violent) | .81** | [.62, .99] |
| START:AV |  |  |
| Violence |  |  |
| Vulnerabilities | .56 | [.38, .75] |
| Strengths^a^ | .68* | [.53, .84] |
| Risk judgement (violence) | .73** | [.58, .88] |
| Suicidal/non-suicidal self-injury |  |  |
| Vulnerabilities | .82*** | [.70, .93] |
| Strengths^a^ | .88*** | [.77, .98] |
| Risk judgement (suicide) | .74* | [.55, .93] |
| Risk judgement (NSSI) | .64 | [.43, .86] |
| VRS-YV |  |  |
| Violence |  |  |
| Static | .49 | [.29, .69] |
| Dynamic | .63 | [.45, .81] |
| Total | .61 | [.42, .80] |
| Suicidal/non-suicidal self-injury |  |  |
| Static | .42 | [.16, .67] |
| Dynamic | .70 | [.47, .93] |
| Total | .66 | [.43, .90] |

*Note.* Prevalence rates for post-baseline violence and suicidal/non-suicidal self-injury are 20.7% and 13.8%, respectively. SRR = Summary Risk Rating; AUC = area under the curve; CI_AUC_ = confidence interval of AUC.

^a^ For ease of interpretation, scores on the Protective/Strengths domains were reversed for the AUC analysis such that higher scores represent a deficit in protective factors.

* *p* < .05, ** *p* < .01, *** *p* < .001 (two-tailed test). Bonferroni correction: *p* = .003.

**Figure S1.** *Cumulative/Dynamic Time-Dependent AUC Analysis Predicting Violence (N =87)***
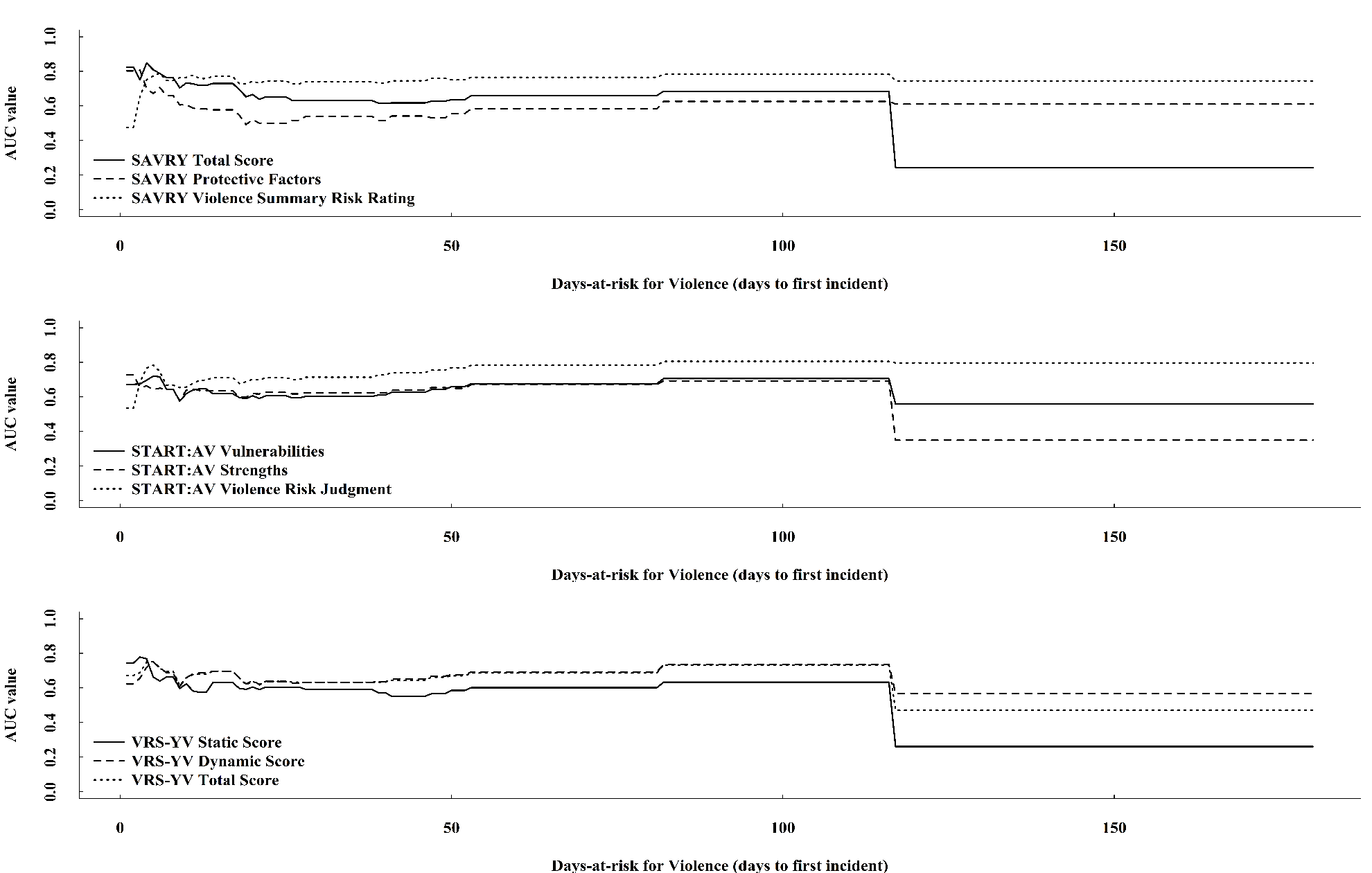
**

*Note.* AUC = area under the curve. For ease of interpretation, scores on the Protective/Strengths domains were reversed for the time-dependent AUC analysis such that higher scores represent a deficit in protective factors/strengths.

**Figure S2.** *Cumulative/Dynamic Time-Dependent AUC Analysis Predicting Suicidal/Non-Suicidal Self-Injury (N =87)*

**
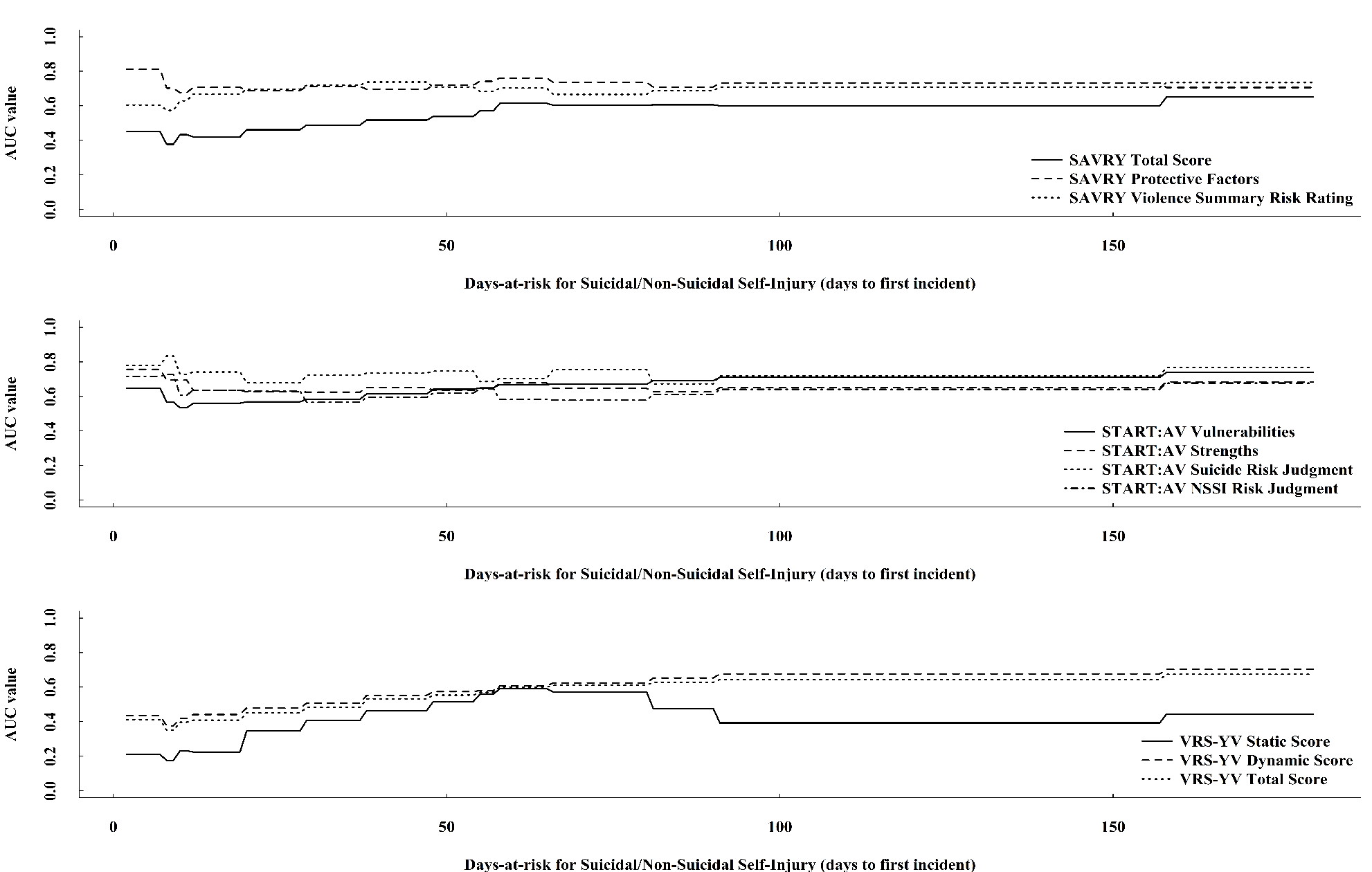
**

*Note.* AUC = area under the curve. For ease of interpretation, scores on the Protective/Strengths domains were reversed for the time-dependent AUC analysis such that higher scores represent a deficit in protective factors/strengths.
